# Supplementary figures and images for: NAVIP: Unraveling the influence of neighboring small sequence variants on functional impact prediction
Source: PLoS Comput Biol. 2025 Feb 18;21(2):e1012732. doi: 10.1371/journal.pcbi.1012732 (PMC11849982; doi:10.1371/journal.pcbi.1012732)

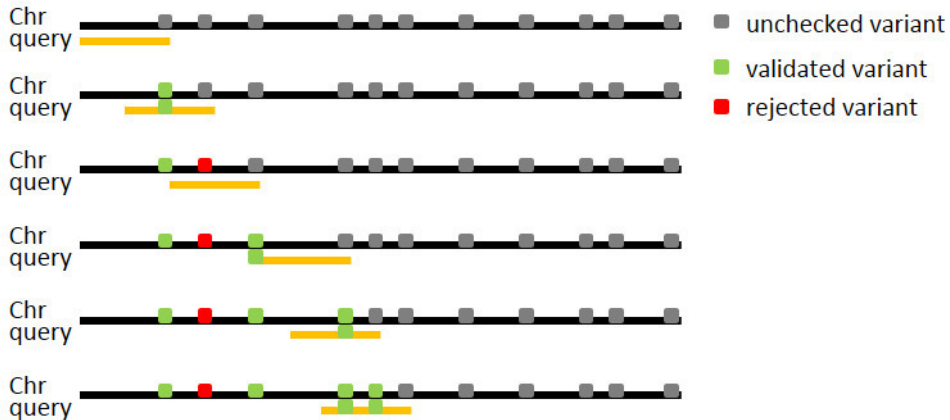

Supplement: S7 File — Schematic illustration of the variant validation process. (PDF) [file pcbi.1012732.s007.pdf]
